# Haemophilia A associated with Down’s syndrome

**DOI:** 10.1007/s00277-012-1619-7

**Published:** 2012-11-14

**Authors:** Barbara Kaczorowska-Hac, Marek Wlazlowski, Jolanta Wierzba, Anna Balcerska

**Affiliations:** Department of Hematology, Oncology and Endocrinology, Medical University of Gdansk, Debinki 7, 80-952 Gdansk, Poland

Dear Editor,

We documented a case of a boy with Down’s syndrome in whom severe haemophilia A was diagnosed. Haemophilia A is an inherited, sex-linked disorder in which coagulation factor VIII (FVIII) is deficient or absent. This disease is the most frequently diagnosed clotting deficiency in newborns occurring at about 1 in 5,000–10,000 male births. Down’s syndrome is a chromosomal condition caused by the presence of all or part of an extra 21st chromosome (trisomy 21), and the incidence of the syndrome is estimated at 1 per 733 births. It is associated with some impairment of cognitive and physical growth and a particular set of facial characteristics. Affected individuals tend to have a lower-than-average cognitive ability, often ranging from moderate to pronounced disabilities.

The patient was a first child born to young parents. His uncle from his mothers’ side also suffered from severe haemophilia A. There were no noted complications during pregnancy and he was delivered in full term. At birth it was noted that the patient presented characteristics of Down’s syndrome. After the administration of hepatitis B vaccination, the patient developed a massive left thigh muscle haematoma. Moreover, he was diagnosed with an atrioventricular canal (AVC/AVSD). His activated partial thromboplastin time was 113 s (reference value 28–36 s) and factor VIII concentration was 0.25 %. Due to the femoral haematoma plasma-derived factor VIII, 30 IU per kg was administered for three consecutive days with improvement. The molecular analysis of haemophilia mutation was not available at the time and karyotype analysis confirmed Down’s syndrome. At 3 months, the boy underwent cardiosurgery without any complications, under plasma-derived FVIII protection. He received a dosage of 50 IU per kg in the first 3 days (including the day the surgery was performed), 40 IU per kg in the next three consecutive days and 30 IU per kg up to the 14th day after surgery with good hemostasis.

He was on-demand plasma-derived FVIII therapy for first 3 years of his life. During that period, he had nine incidents of soft tissue bleeding after slight injuries (gum, calf, chest muscles). The dosage of factor VIII concentrate was 2,400 IU per year. At the age of 3, after his first episode of hip joint bleeding, he started prophylaxis with plasma-derived factor VIII but due to limitation of FVIII prophylaxis accessibility in our country, therapy was halted after 2 months of administration. During the on-demand therapy which was administrated till he was 5 years, the boy presented with eight incidents of joint bleeding (hip, knee, ankle). The dosage of FVIII was 11,500 IU per a year. Ultimately the boy restarted prophylaxis at the age of 5 when national haemophiliacs plasma-derived FVIII prophylaxis programme started in our country. On the primary dose of 20 IU per kg twice a week, a knee bleeding was noted and the dosage was increased to 40 IU per kg three times weekly. In 3 years of high-dose prophylaxis, he did not have any incidents of joint or soft tissue bleeding. Regular tests excluded development of FVIII inhibitor.

Currently at age of 8, the patient is still continuing with prophylaxis. He attends school activities and participates in rehabilitation exercises.

Clinical and numerous other studies of haemophiliacs demonstrate that primary prophylaxis is much superior to on-demand treatment [1–3]. Unfortunately, prophylaxis accessibility in many health services is limited.

We presented management of a boy with Down’s syndrome and severe haemophilia A. Replacement of FVIII provided successful concomitant heart defect cardiosurgery [4]. Due to our national health programme, the boy was on-demand plasma-derived FVIII therapy. For the first 2 years, he had a few symptoms due to delayed crawling, inability to walk and great parental overprotectiveness. Frequency of bleeding incidences increased with time as he became more active. At age of 5 he was entered into the national health prophylaxis programme. The decision to start him on this regimen was discussed with the patient’s psychologist in order to decide whether both the patient and his parents would cope with regular injections. Home therapy was found to be feasible due to appreciable parental commitment. The administration of high-dose secondary prophylaxis resulted in marked improvement with no bleeding episodes noted. Regardless of the fact that secondary prophylaxis was delayed, the benefit of the regimen used in a handicapped boy was unquestionable.

To our knowledge, this is the second case in the available literature, describing the association of haemophilia A and Down’s syndrome [5] and the first report of a prophylaxis regimen in coexistence of these two diseases.
